# Can public consultation effectively optimise the design of a patient information leaflet about breast magnetic resonance imaging?

**DOI:** 10.1186/bcr3775

**Published:** 2015-11-05

**Authors:** Lyn Jones, Anna Mankelow, Joanne Robson, Anjum Mahatma, Alice Pocklington, Alexandra Valencia

**Affiliations:** 1 Bristol Breast Care Centre at North Bristol NHS Trust, Bristol, UK

## Introduction

Breast magnetic resonance imaging (MRI) involves multiple aspects that are unique to a medical environment and may seem frightening and strange to a person from a non-medical background (the tunnel, no credit cards, keys or watches, loud noises, intravenous pump injector). The purpose of an information leaflet is to inform people about what they should expect, and to prepare them for the experience. During public consultation about breast MRI, we discovered that women considered the current information provided by the NHS (from several different hospitals) to be inadequate. They told us that their experience of the process of breast MRI had been more distressing that it would have been had they been better informed. We decided to ask their advice on the design of an information leaflet to see if it could be optimised to better prepare women for the experience.

## Methods

Public consultation was used to identify aspects of breast MRI that required explanation in an information leaflet and how they would like the information presented. We incorporated their suggestions into our new design and asked for comments at a second public consultation session.

## Results

The need for intravenous access, the tunnel, the nature of the loud noises that changed during the scan and knowledge that the radiographers could see and hear them throughout the scan were all emphasised as requiring explanation. The public suggested the use of multimedia including links to videos, sounds and personal accounts of experience.

## Conclusion

Our new leaflet has been approved by the public and patients.

